# Structured Program for Weight Loss in Heart Failure Patients: Findings From a Cardiology-Driven Obesity Clinic

**DOI:** 10.1016/j.jacadv.2023.100741

**Published:** 2023-11-30

**Authors:** Vijay U. Rao, Kaitlin Ziedonis, David Gunderman, Ashish Kumar, Anthony Bashall, Kathy Stark, Atul R. Chugh, Ryan Daly, Ankur Kalra

**Affiliations:** a Franciscan Health, Indianapolis, Indiana, USA; b Indiana University School of Medicine, West Lafayette, Indiana, USA; c Cleveland Clinic Akron General, Akron, Ohio, USA; d Franciscan Health, Lafayette, Indiana, USA; e Krannert Cardiovascular Research Center, Indianapolis, Indiana, USA

The escalating prevalence of obesity, a critical risk factor for heart failure (HF) onset and progression, highlights the necessity for strategic weight loss interventions in general cardiology and HF clinic settings. Obesity represents a major risk factor for HF; each 1-U increase in body mass index (BMI) potentiates HF risk by 5% and 7% in men and women, respectively.^1^ Evidence of the effectiveness of glucagon-like-peptide (GLP)-1 and glucose-dependent insulin tropic polypeptide (GIP)/GLP-1 agonists for weight loss in HF patients with obesity has recently received more attention, with a recent randomized controlled trial reporting an average weight loss of −13.3% after 52 weeks of semaglutide treatment with better outcomes among this patient population.^2^ In 2022, at Franciscan Health, Indianapolis, an Obesity Task Force was commissioned to tackle obesity by surveying existing local resources, developing clinical pathways, and instituting a cardiology-driven obesity clinic for our outpatient HF patients. Further, while the effectiveness of GLP-1 or GIP/GLP-1 drug classes in clinical settings has been studied in patients with obesity,^3^^,^^4^ no study has shown the generalizability of these drugs to real-world clinical settings for HF patients.

**Figure 1:**
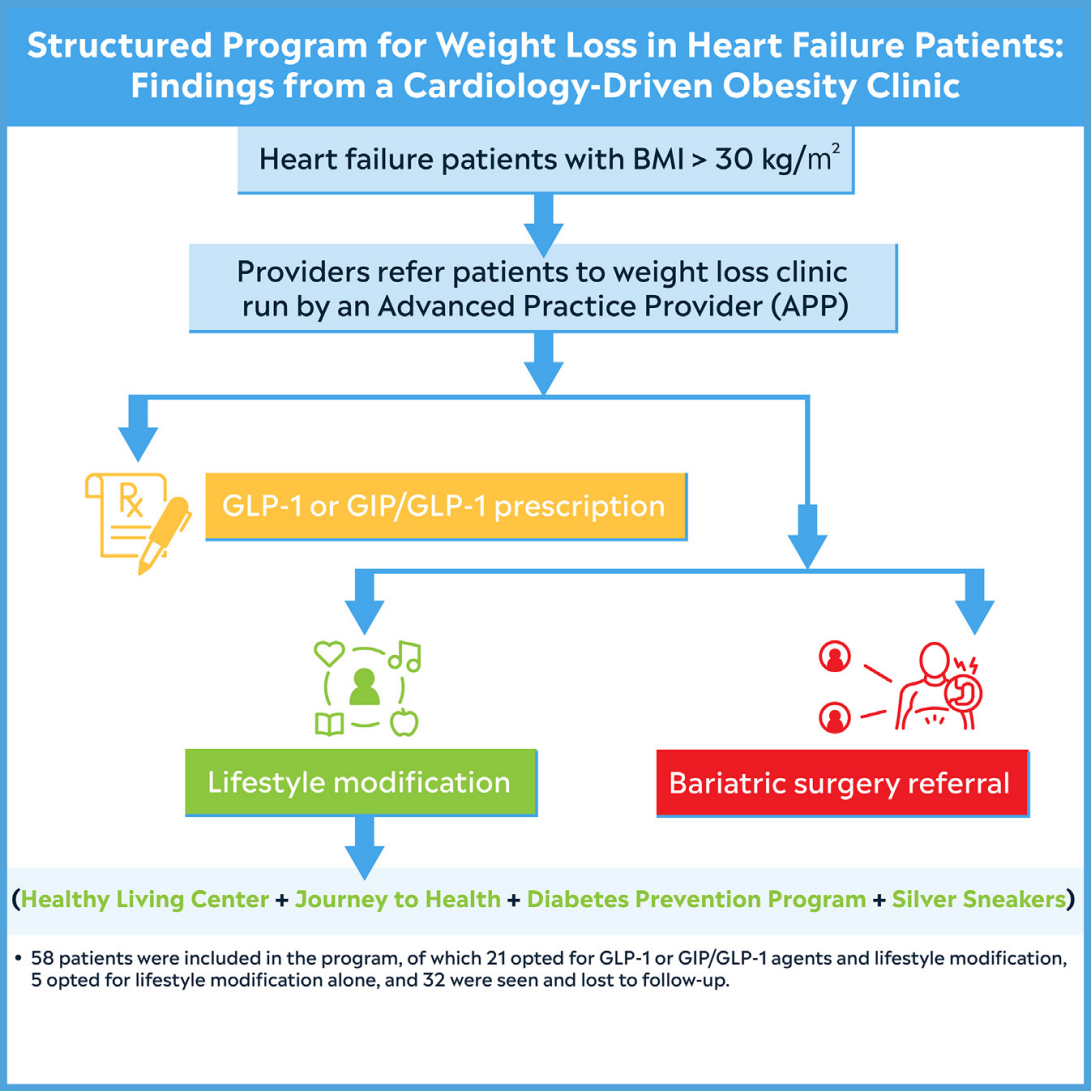
**HF Obesity Algorithm Within the Setting of a HF Clinic** Glucagon-like-peptide-1 (GLP-1) and glucose-dependent insulin tropic polypeptide (GIP)/GLP-1. HF = heart failure.

A collaborative team of experts spanning cardiology, endocrinology, bariatric surgery, primary care, nurses, health coaches, and lifestyle program administrators developed and implemented a HF obesity algorithm within the setting of a HF clinic, as depicted in Figure 1. Patients with an established HF diagnosis, a BMI ≥30 kg/m^2^, and a willingness to participate in lifestyle modifications were identified in the inpatient and outpatient setting and referred to a designated HF advanced practice provider for outpatient care. In brief, the initiatives of the lifestyle modification programs were; major initiatives of *Healthy Living Center*: one-on-one coaching from advanced practice registered nurse and personalized plan for weight loss counseling, lifestyle modifications, and use of intensive-behavioral therapy for weight loss management. There was monitoring and follow-up visits every 2 to 4 weeks. Major initiatives of *Journey to Health* included a yearlong program of combination visits with primary care physicians and certified health coaches, nutrition and weight loss program, tracking of body composition with DXA scans, incorporation of *diabetes prevention program* if applicable, group cooking classes, and group fitness classes. Major initiatives of cardiac rehabilitation included qualifying patients (postmyocardial infarction, HF with reduced ejection fraction [HFrEF]), individualized exercise plan (aerobic, strength training, flexibility), outpatient education by dieticians, exercise physiologists, pharmacists, and diabetes educators. Major initiatives of *Silver Sneakers* included a fitness and wellness program designed for seniors 65 and older on certain Medicare programs, in-person and virtual options with gym access to participating locations, cardio equipment, pool activities, tennis, and walking tracks with online resources on nutrition and fitness. Utilizing a shared decision-making process including discussion of cost of GLP-1 or GIP/GLP-1 agents, patients were started on GLP-1 or GIP/GLP-1 agonists, primarily incorporating semaglutide or tirzepatide, with the choice influenced by diabetic status. Over a period ranging from 6 weeks to 10 months, patients were periodically assessed to fine-tune medication dosages and monitor progress. Compliance was determined by regular follow-ups and appointments for refill prescriptions. The current study was deemed exempt from the Franciscan Health institutional review board approval. Continuous variables were presented as median (range), while categorical variables were presented as absolute numbers (IQR). All statistical analyses were carried out using R version 4.0.3.

Fifty-eight patients were included in the program, of whom 21 opted for GLP-1 or GIP/GLP-1 agents and lifestyle modification, 5 opted for lifestyle modification alone, and 32 were seen and lost to follow-up considering the cost of the GLP-1 or GIP/GLP-1 agents, and they did not want to participate in lifestyle modification. The study sample prescribed GLP-1 or GIP/GLP-1 agents (n = 21) had a median age of 57 (IQR: 39-81) years, a median starting weight of 295 (IQR: 214-441) lbs, and a median starting BMI of 43.65 (IQR: 32.35-63) kg/m^2^. The median follow-up duration was 182 (IQR: 75-268) days. None of the 21 patients were on any other weight-reduction medications. Of the 21 participants, 12 (61.9%) were female, 17 (80.9%) had HF with preserved ejection fraction, 4 (19.05%) had HFrEF, and 10 (47.6%) had pre-existing diabetes. Of the 4 HFrEF, 2 were ischemic and 2 were nonischemic. The median weight loss among the HF patients on any GLP-1 or GIP/GLP-1 was 21 (IQR: 6-63) lbs, or −9.4% (IQR: −2.3% to −26.4%).

Addressing obesity in the HF population represents a significant unmet medical need. Here, we describe a successful approach to tackling this important risk factor through implementation of a cardiology-driven obesity clinic for our outpatient HF patients. Further, in the brief time since implementation, we have seen clinically significant mean weight loss among patients who were prescribed GLP-1 or GIP/GLP-1 agents. The major limitation of the study is the observational nature of the study design with possibility of selection bias and a small sample size. A wide range of follow-up duration adds to the limitation of the study. However, to our knowledge, this is the first study to show that GLP-1 and GIP/GLP-1 treatment in a HF clinic setting is associated with weight loss similar to that seen in randomized clinical trials, suggesting the applicability of these drugs in treating patients with concurrent HF and obesity in the clinical setting.
